# 172 The Effects of Antenatal Behaviour-Change Lifestyle Interventions on Improving Maternal and Foetal Health Outcomes: A Systematic Review and Meta-Analysis Protocol

**DOI:** 10.1093/eurpub/ckae114.188

**Published:** 2024-09-26

**Authors:** Abbey O’Keeffe, Elizabeth Deery, Sinéad Currie, Gemma McMonagle, Maria Faulkner

**Affiliations:** Department of Law & Humanities, Faculty of Business, Atlantic Technological University, Donegal, Letterkenny, Ireland; SPHeRE PhD Programme, School of Population Health, Royal College of Surgeons in Ireland, Dublin, Ireland; School of Sport, Faculty of Life & Health Sciences, Ulster University, Derry-LondonDerry, Northern Ireland; Department of Psychology, Faculty of Natural Sciences, University of Stirling, Stirling, Scotland; Department of Health & Nutritional Sciences, Faculty of Science, Atlantic Technological University, Sligo, Sligo, Ireland; Department of Law & Humanities, Faculty of Business, Atlantic Technological University, Donegal, Letterkenny, Ireland

## Abstract

**Purpose:**

The purpose of this systematic review is to examine the effects of antenatal behaviour-change lifestyle interventions that focus on physical activity (PA) and evidence-based nutritional practices (NP) to improve maternal and foetal outcomes, and pregnant individuals’ adherence to lifestyle interventions.

**Methods:**

The following databases will be searched from their inception using a detailed search strategy, MEDLINE; CINAHL; PsycINFO; Embase; AMED; WHOLiS, Web of Science; ScieLo and ASSIA. A Population, Intervention, Comparator, Outcome and Study-Design framework will guide the selection process. The review will be restricted to Randomised Controlled Trials that compared the effects of nonpharmacological, antenatal behaviour-change PA and NP lifestyle interventions. The review will utilise the FITT (Frequency, Intensity, Time/Duration and Type) principle of PA and an adapted-NP classification table to categorise interventions. Interventions will be evaluated using the ‘Framework for Developing and Evaluating Complex Interventions’.

Primary outcomes will include maternal and foetal health outcomes, including: maternal mental health-status; gestational weight-gain; gestational diabetes mellitus diagnosis; mode of delivery; birthweight; gestational age and perinatal mortality. Secondary outcomes will include adherence to interventions. Authors will independently screen titles, abstracts, and full-text articles according to inclusion/exclusion criteria. A standardised data extraction tool will be developed based on the Cochrane Handbook of Systematic Reviews of Interventions. The Cochrane Collaboration’s Risk of Bias Assessment tool will assess methodological quality of studies and GRADEpro criteria will evaluate the quality of the evidence.

**Results:**

Meta-analysis will be employed to synthesise the results on the effects of the interventions on maternal and foetal outcomes. Primary outcomes will be pooled using a random-effects model. Subgroup analyses will explore variations across participant characteristics or intervention types. Sensitivity analyses will assess the impact of missing data. Heterogeneity among studies will be assessed quantitatively using statistical tests and the I^2^ statistic, with planned exploration of potential sources of heterogeneity through subgroup analyses, followed by meta-regression.

**Conclusions:**

This review will synthesise available evidence on the effectiveness of antenatal behaviour-change lifestyle interventions to provide valuable information for clinicians and health policymakers to improve policy and practice.

**Support/Funding Source:**

Atlantic Technological University, Donegal Research Bursaries and The Health Research Board’s SPHeRE PhD Programme.

